# Shiga Toxin Mediated Neurologic Changes in Murine Model of Disease

**DOI:** 10.3389/fcimb.2016.00114

**Published:** 2016-09-29

**Authors:** Suman Pradhan, Christine Pellino, Kayleigh MacMaster, Dennis Coyle, Alison A. Weiss

**Affiliations:** ^1^Department of Molecular Genetics, Biochemistry and Microbiology, University of CincinnatiCincinnati, OH, USA; ^2^KAO USA Inc.Cincinnati, OH, USA

**Keywords:** bacterial toxin, H&E staining, microglia activation, Iba1 antibody, mouse brain

## Abstract

Seizures and neurologic involvement have been reported in patients infected with Shiga toxin (Stx) producing *E. coli*, and hemolytic uremic syndrome (HUS) with neurologic involvement is associated with more severe outcome. We investigated the extent of renal and neurologic damage in mice following injection of the highly potent form of Stx, Stx2a, and less potent Stx1. As observed in previous studies, Stx2a brought about moderate to acute tubular necrosis of proximal and distal tubules in the kidneys. Brain sections stained with hematoxylin and eosin (H&E) appeared normal, although some red blood cell congestion was observed. Microglial cell responses to neural injury include up-regulation of surface-marker expression (e.g., Iba1) and stereotypical morphological changes. Mice injected with Stx2a showed increased Iba1 staining, mild morphological changes associated with microglial activation (thickening of processes), and increased microglial staining per unit area. Microglial changes were observed in the cortex, hippocampus, and amygdala regions, but not the nucleus. Magnetic resonance imaging (MRI) of Stx2a-treated mice revealed no hyper-intensities in the brain, although magnetic resonance spectroscopy (MRS) revealed significantly decreased levels of phosphocreatine in the thalamus. Less dramatic changes were observed following Stx1 challenge. Neither immortalized microvascular endothelial cells from the cerebral cortex of mice (bEnd.3) nor primary human brain microvascular endothelial cells were found to be susceptible to Stx1 or Stx2a. The lack of susceptibility to Stx for both cell types correlated with an absence of receptor expression. These studies indicate Stx causes subtle, but identifiable changes in the mouse brain.

## Introduction

Disease due to Shiga toxin (Stx)-producing *Escherichia coli* (STEC) is a significant cause of foodborne illness, with an estimated 265,000 cases annually (Scallan et al., 2011). In addition to hemorrhagic colitis, the systemic complication, hemolytic uremic syndrome (HUS), occurs when the protein toxin, Stx gains access to the circulation. Stx is an AB_5_ toxin, and consists of a single A-subunit of 32 kDa and 5 identical B-subunits of 7.7 kDa (Donohue-Rolfe et al., 1984). The A-subunit is an enzymatically active N-glycosidase that inhibits protein synthesis by removing a single adenine at position 4324 from the 28S rRNA of the eukaryotic ribosomal 60S subunit (Endo et al., 1988). The B-pentamer promotes cell-association by binding to globotriaosyl ceramide (Gb3) expressed on mammalian cells and delivery of the A-subunit to the cytoplasm. Recent studies have shown that A- and B-subunit assembly occurs on the surface of cells (Pellino et al., 2016). Stx includes two major antigenic forms, Stx1 and Stx2, which share approximately 60% amino acid identity (Strockbine et al., 1986), and subtypes of Stx2 (a–h) can share >90% amino acid identity (Scheutz et al., 2012). Epidemiological and molecular typing studies indicate that strains producing Stx2 subtype a (Stx2a) are most commonly associated with life-threatening human disease (Ostroff et al., 1989; Boerlin et al., 1999; Eklund et al., 2002; Persson et al., 2007).

Damage to the vasculature and kidney play a prominent role in the development of HUS (Tarr et al., 2005). Platelet thrombus formation in the microvasculature compromises blood flow to the kidney. Hemolytic anemia develops when red blood cells are mechanically sheared as they squeeze through the occluded vessels. In addition, neurologic complications are also seen in HUS, and include movement disorders, diplopia, dysphasia, facial palsy, alteration in consciousness, seizures, and coma (Cimolai and Carter, 1998; Magnus et al., 2012; Trachtman et al., 2012). HUS with neurologic involvement is associated with more severe outcome. In the Germany outbreak in 2011, 48% of the hospitalized patients in Germany developed severe neurological symptoms (Magnus et al., 2012), some were readmitted to the hospital after kidney damage had resolved (Jansen and Kielstein, 2011).

The molecular basis for neurologic symptoms during STEC infection is unclear. There is little evidence of cellular death in the brain, and permanent neurologic damage is typically not observed in human patients after resolution of the acute symptoms. Evidence of neurologic involvement has been reported in Stx2-treated mice, and like humans, mice display little evidence of gross cellular damage. In this study, we monitored Stx-treated mice treated at doses that induce injury to the kidney for evidence of neurologic damage using histologic examination and non-invasive MRI. Microglial cells were examined as early indicators of neural injury (Kreutzberg, 1996). Due to their distribution and morphology, microglia are in constant and intimate contact with multiple signals originating from nearby neurons and macroglia. Responses to damage can include up-regulation of surface marker expression (e.g., Iba1) and stereotypical morphological changes from the ramified morphology of “resting” microglia to the “activated” macrophage-like state (Ito et al., 1998; Imai and Kohsaka, 2002). Subtle changes in the brains of the mice were seen using both MRS and histology.

## Materials and methods

### Bacterial toxins

Purified recombinant Stx1 (cat. # NR-857) and Stx2a (cat. # NR-4478) were obtained from BEI resources. Stx was diluted in tissue culture grade PBS (pH 7.4) for all inoculations. Lipopolysaccharide (LPS) content was determined by the limulus amoebocyte lysate (LAL) assay (Lonza).

### Mouse studies

All animal studies were approved by the Institutional Animal Care and Use Committee (IACUC) of the University of Cincinnati, and conducted in strict accordance with the recommendations of the Guide for the Care and Use of Laboratory Animals. Outbred male CD-1 mice, 13–15 g obtained from Charles River Laboratories (Wilmington, MA) were housed in filter-top cages with access to food and water *ad libitum*. Mice were challenged by intraperitoneal (IP) injection. Mice were observed twice daily for signs of illness, and weighed once a day.

### Brain and kidney histology

Mice were given Stx2a at 7 ng/ml and Stx1 at 1500 ng/ml. PBS alone served as the negative control. At the indicated times post-challenge, animals were anesthetize by IP injection of 100 μl, 2.5% avertin (2,2,2-Tribromoethanol) and perfused intracardially with 100 ml sterile PBS containing 20 units/ml heparin sodium salt to prevent clotting. Organs were fixed by perfusion with 200 ml of 4% paraformaldehyde prepared by dissolving 20 g of paraformaldehyde powder in 500 ml of heated PBS solution, cooled, adjusted to pH 6.9 and filter sterilized. After perfusion, organs were removed and stored in 4% paraformaldehyde.

For brain histology studies, the tissues were washed in PBS with 3 changes over a period of 15 min and embedded in 5.0% low gelling temperature agarose (Sigma Type XI, A-3038) in 20 mm peel-a-way disposable plastic tissue embedding molds (Thermo Scientific, cat no: 2219). Forty micron thick brain samples were sectioned on the vibratome and stored in 4% paraformaldehyde until stained. For kidney histology, thin sections of 5 micron thickness were cut using Leica CM 1900 cryostat and mounted on slide until stained. For hematoxylin and eosin staining, brain and kidneys were processed by fixing in 4% paraformaldehyde solution, and then dehydrated in graded alcohol, cleared by xylol and embedded in paraffin.

### Microglial staining

Rabbit polyclonal antibody against Iba1 (ionized calcium binding adaptor molecule-1) was used as the marker for microglial detection. The protocol was followed as laid down in the Vectastain Elite ABC system kit from Vector Laboratories. Briefly, brain sections were incubated with blocking buffer (1.5% normal goat serum (Vector Laboratories, cat no: S-1000) and 1% Bovine Serum Albumin in 1X PBS) for 2 h at room temperature (RT) followed by incubation with anti-Iba1 antibody, 0.5 μg/ml (Wako Catalog No. 019-19741) in blocking buffer (1.5% normal goat serum (Vector Laboratories, cat no: S-1000) and 1% Bovine Serum Albumin in 1X PBS) overnight at 4°C. The sections were washed with 1X PBS, 3 times and incubated with Biotinylated anti-Rabbit IgG Antibody (1:200) in blocking buffer for 1 h at RT and further washed with 1X PBS, 3 times prior to incubation with Vectastain Elite ABC (Reagent A (1:50) and Reagent B (1:50) in 0.01M PBS) for 1 h at RT. The sections were washed three times and incubated in peroxidase solution (0.01% hydrogen peroxide and 0.05% DAB (3, 3′-diaminobenzidine) HRP substrate in 0.05 M Tris buffer) for color development.

### Magnetic resonance spectroscopy (MRS)

Forty eight to seventy two hours after IP injection with Stx2a (3 ng) or PBS animals were transferred to Cincinnati Children's Hospital and Medical Center, Department of Radiology, Imaging Research Center. All of the Stx2a-treated mice were symptomatic at the time of imaging, as evidenced by lack of weight gain, weight loss and/or lethargy, and the mice that were most severely affected were imaged first. For the toxin-treated animals, two were imaged on day 2, and two were imaged on day 3. For the control animals, two were imaged on day 2 and one was imaged on day 3. Animals received standard MRI brain scans and MRS data was collected from the thalamus and cortex. Unprocessed MRS signals were analyzed using the LCModel software package (version 6.2-0), data where the % standard deviation exceeded 15% were rejected.

### Statistical analysis

Images of immunohistochemically developed slides were captured using an Aperio ScanScope slide scanner (Aperio, Vista, CA) to create whole-slide digital images. Images were subsequently analyzed using ImageScope Positive-Pixel Count Algorithm (Aperio) for systematic identification and quantitation of deposited DAB generating a pseudo-colored markup image representing staining intensities. Staining intensities were assigned using as parameters the following selections for input upper limits: weak positive (70, yellow on markup image), medium positive (100, orange) and strong positive (180, brown). This set of algorithm settings was used on all positive and negative control tissues with manual comparisons of multiple fields to ensure validity of the selected settings. Analysis of variance was used to determine the significant difference in the survival curve among intoxication groups as well as for the brain microglial study followed by Bonferroni post-tests (GraphPad Prism 5, GraphPad Software, Inc.). *P*-values < 0.05 were considered significant.

### Endothelial cell culture

Human cerebral cortex microvascular endothelial cells (HBMEC, ACBRI 376, Lot 376.04.0H.0U.0Y) were obtained from Cell Systems Corporation (Kirkland, WA) and were propagated in Complete Classic Medium with serum, CultureBoost and Bac-Off® antibiotic (Cell Systems, Kirkland, WA). Cells were harvested with the Passage Reagent System (Cell Systems) according to manufactures protocol. The BALB/c bEnd.3 cell line was a gift from Dr. Jerry Lingrel and was maintained in DMEM with 10% FBS and Pen/Strep. Primary neonatal dermal microvascular endothelial cells (dHMEC, CC-2516, Lot 0000317328) were obtained from Clonetics (Lonza, Walkersville, MD) and propagated in Endothelial Cell Medium without phenol red (ScienCell, Carlsbad, CA). Cells were harvested with TrypKit (Lifeline Cell Technologies, Walkersville, MD) according to manufacturer's protocol. The CDC.HMEC-1 line was maintained in MCDB 151 medium supplemented with 10 mM L-glutamine, 10 ng/ml epidermal growth factor, 1 μg/ml hydrocortisone, 10% fetal bovine serum (FBS), 50 μg/ml gentamycin sulfate and 100 μg/ml kanamycin sulfate.

### Endothelial cell toxicity assay

Stx was serially diluted in 20 μl of medium in sterile, black, clear, flat-bottom 96-well plates (Corning, Tewksbury, MA). Cells (in 60 μl of medium) were added to toxin-containing wells at subconfluent or confluent numbers, as follows: BMEC (subconfluent 1.5 × 10^3^ cells per well, confluent 8 × 10^3^ cells per well), bEnd.3 and dHMEC (subconfluent 6 × 10^3^ cells per well, confluent 12 × 10^3^ cells per well). Toxin-treated cells were incubated at 37°C in 5% CO_2_. After 42 h the medium was removed and 50 μl of fresh medium containing 10% (vol/vol) alamarBlue (AbD Serotec, Raleigh, NC) was added. Cells were incubated in the presence of alamarBlue for a total of 3 h. Fluorescence was read at 590 nm on a FLX-800 fluorimeter (BioTek, Winooski, VT) every 30 min and values within the linear range, before depletion of substrate, are reported. In assays using TNF-α, cells were plated at the subconfluent or confluent densities in 60 μl of medium and incubated overnight. TNF-α (10 ng/ml) was added the next day and 24 h later toxin was added. Cells were incubated for an additional 42 h and analyzed as described above. Dose-response curves were plotted as the percent relative fluorescence units (RFU) of untreated cells verses toxin concentration.

### Flow cytometry to detect Gb3

5 × 10^4^ cells were cooled on ice for 30 min and all subsequent incubations were carried out on ice. Cells were incubated for 1 h with antibody to Gb3 (clone 38-13, Accurate Chemical and Scientific Corporation, Westbury, NY) or PBS for controls. Cells were washed, incubated for 1 h with fluorescein isothiocyanate (FITC) conjugated α-rat IgM secondary antibody diluted 1:500 (AbD Serotec, product number MCA189F, Bio-Rad, Raleigh, NC) and analyzed by flow cytometry (BD FACSCalibur; Becton Dickinson, Franklin Lakes, NJ). Due to the limited number of cells, analysis was performed by collecting 1 × 10^4^ events or all events collected for 5 min. Unstained cells (for labeled primary antibody) or cells incubated with secondary antibody (for unlabeled primary antibody) were used as negative controls. Cells were gated for the live cell population based on the forward and side scatter. Statistical analysis for flow cytometry was performed by paired Student's *t*-test using Prism5 (GraphPad Software, La Jolla, CA).

### Pretreatment with TNF-α

To assess the role of TNF-α on Gb3 expression, cells were plated in 12-well plates and allowed to adhere overnight. TNF-α (10 ng/ml) was added and 24 h later cells were harvested and treated as described above. As a positive control for TNF-α activity, stimulated HBMECs were stained for induction of ICAM-1 expression and analyzed by flow cytometry. Allophycocyanin (APC) labeled ICAM-1 (CD54) antibody was diluted 1:200 (clone HA58, product number 353111, BioLegend, San Diego, CA).

## Results

### Weight loss in Stx-treated mice

**Figure 1:**
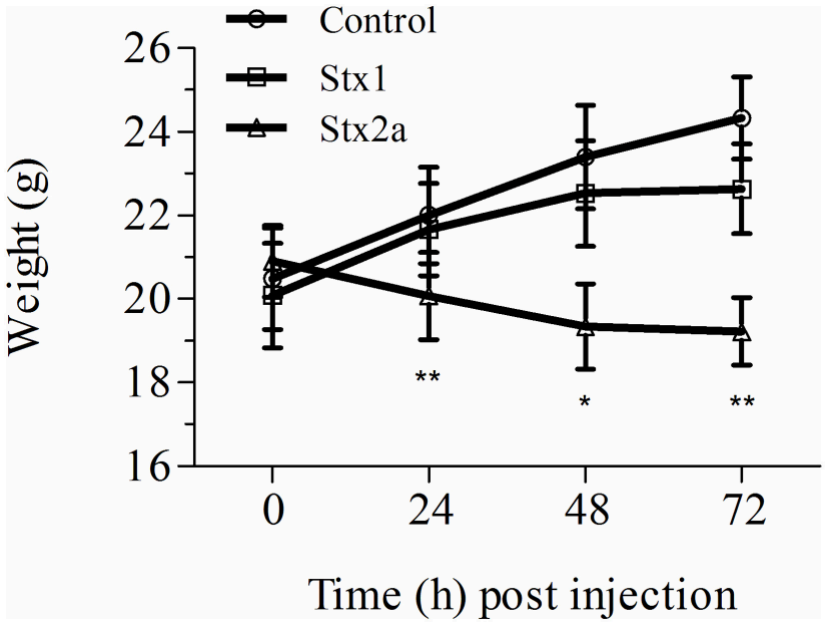
**Weight gain in Stx-treated mice**. Three groups of initially 16 mice were injected with either PBS (control), Stx1 (1500 ng), or Stx2a (7 ng), and weighed prior to sacrificing four mice every 24 h for histological studies. The average weight of the remaining mice at each time point is plotted. As observed in previous studies, mice injected with PBS (circles) gained weight over the 72 h period, while injection of Stx2a (triangles) resulted in a statistically significant loss of weight at 24, 48, and 72 h post-injection (^*^P < 0.01, ^**^P < 0.0032). Compared to the PBS alone injected population, mice injected with Stx1 (squares) gained less weight, although the differences were not statistically significant.

Stx toxicity was validated using standard assays. Groups of mice were injected IP with PBS, purified Stx1 (1500 ng) or purified Stx2a (7 ng), and weighed every 24 h post-injection (Figure 1). Mice injected with PBS gained weight over the 72 h period (Figure 1, closed circles). As observed previously, injection of 7 ng of Stx2a resulted in greater than 50% mortality (Fuller et al., 2011) and statistically significant weight loss was observed (Figure 1, closed triangles). In this study injection of 1500 ng of Stx1 did not result in statically significant weight loss (Figure 1, closed squares). These results demonstrate that Stx1 is not as toxic as Stx2a. Stx2a, the more important toxin in human disease, was the primary focus for subsequent studies.

### Kidney damage in Stx-treated mice

**Figure 2:**
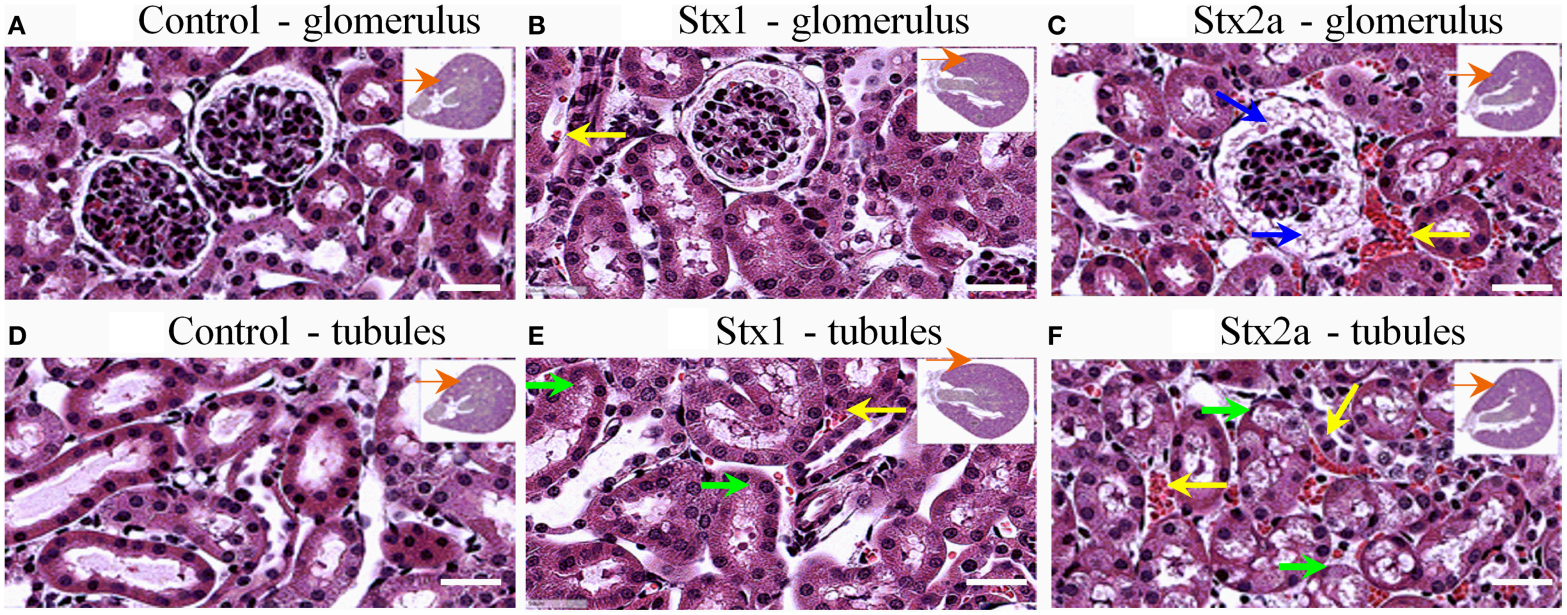
**H&E staining of kidney tissue at 72 h post-injection**. Transverse sections of kidney tissue showing glomeruli **(A–C)** and tubules **(D–F)** of mice from Figure 1 injected with: PBS **(A,D)**; 1500 ng Stx1 **(B,E)**; 7 ng Stx2a **(C,F)**. Stx2a-treated mice show increased spacing in the Bowman's capsule (**C**, blue arrow). Red blood cell congestion is seen in the Stx2a-treated mice (**C,F**, yellow arrows) and to a lesser extent in the Stx1-treated mice (**B,E**, yellow arrows), but not in the kidneys of control mice. Tubular necrosis was seen in animals receiving Stx1 (**E**, green arrows), but these lesions were much more prominent in animals receiving Stx2a (**F**, green arrows). Scale bar represent 50 μm. Insert, orange arrow indicates approximate position of the magnified image.

Kidney damage was assessed at 72-h post-injection (Figure 2). Red blood cell congestion was observed in the Stx1 and Stx2a-treated mice (Figure 2, yellow arrows), but not the control mice. The failure of extensive perfusion in the presence of the anticlotting agent, heparin, to eliminate the red blood cells from the tissues suggests the presence of preexisting clots. Increased spacing in the Bowman's capsule was observed in the glomeruli of the Stx2a-treated mice (Figure 2C, blue arrows). In addition, the Stx2a-treated mice displayed diffuse tubular dilation in the renal cortexes, and minimal-to-moderate acute tubular necrosis of distal tubules, characterized by tubules lined with degenerating, necrotic, or sloughed epithelial cells (Figure 2F, green arrows). Kidney lesions were present in the Stx1-treated mice (Figures 2B,E, yellow and green arrows), but were less severe than those observed in the Stx2a treated animals. Thus, as observed in previous studies, Stx2a is more potent than Stx1, with the smaller dose of Stx2a (7 ng) causing greater weight loss and kidney pathology than the much larger dose of Stx1 (1500 ng).

### Histologic changes in the mouse brain

**Figure 3:**
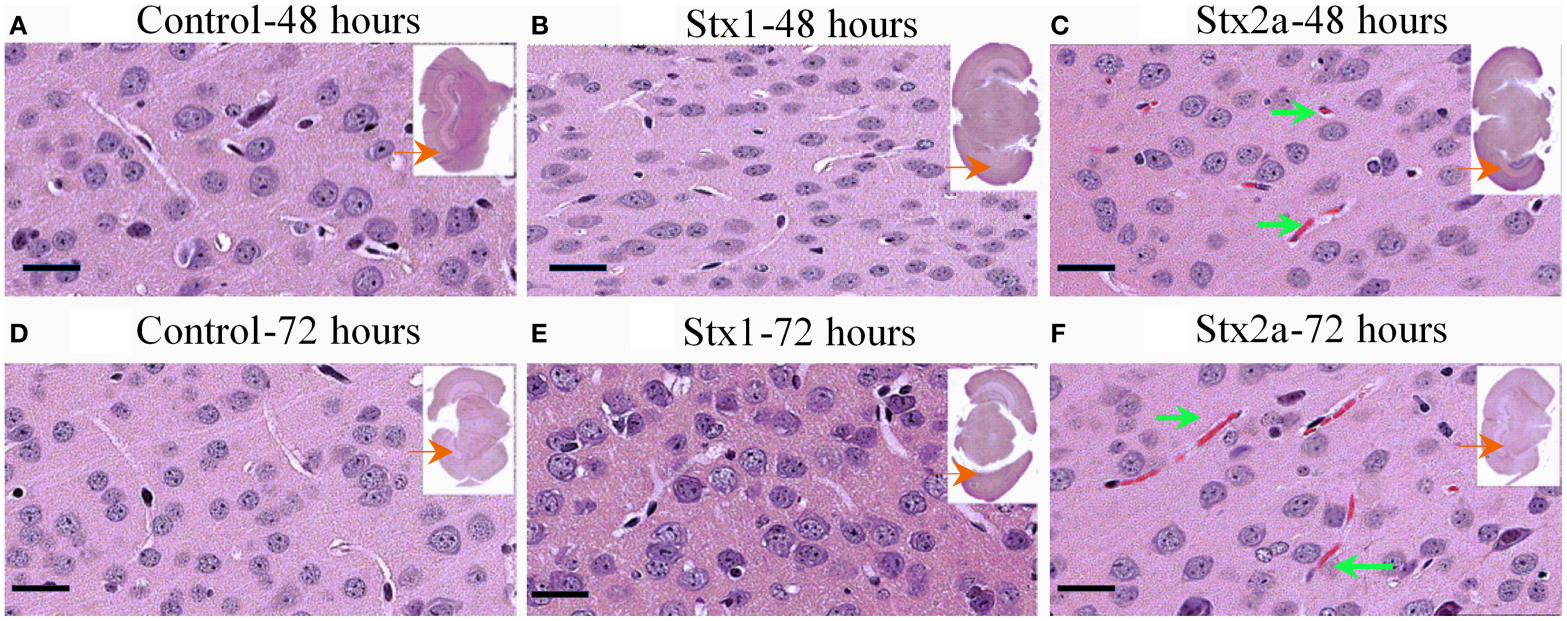
**H&E staining of coronal sections of brain tissue**. Mice from Figure 1 injected with PBS **(A,D)**; 1500 ng Stx1 **(B,E)**; 7 ng Stx2a **(C,F)** sacrificed at the indicated times. Sections of the Stx2a injected mice show accumulation or congestion of red blood cells in the vesicles (**C,F**, green arrows), not seen in the sections injected with PBS alone or Stx1. Scale bar represent 50 μm. Insert, entire brain scan where black arrow indicates approximate position of the magnified image.

Coronal sections of mouse brain tissue were stained with H&E (Figure 3), and few changes were observed. However, as observed in the kidney, red blood cells were present in the brains of the Stx2a-treated mice. They first appeared at 24 h post-injection (data not shown), and at 48 and 72 h post-injection, large areas of red blood cell congestion and clumping were evident throughout the cortex (Figures 3C,F, green arrows). RBC congestion was not observed in the brains of the Stx1-treated or control mice.

### Stx causes microglial cell activation

**Figure 4:**
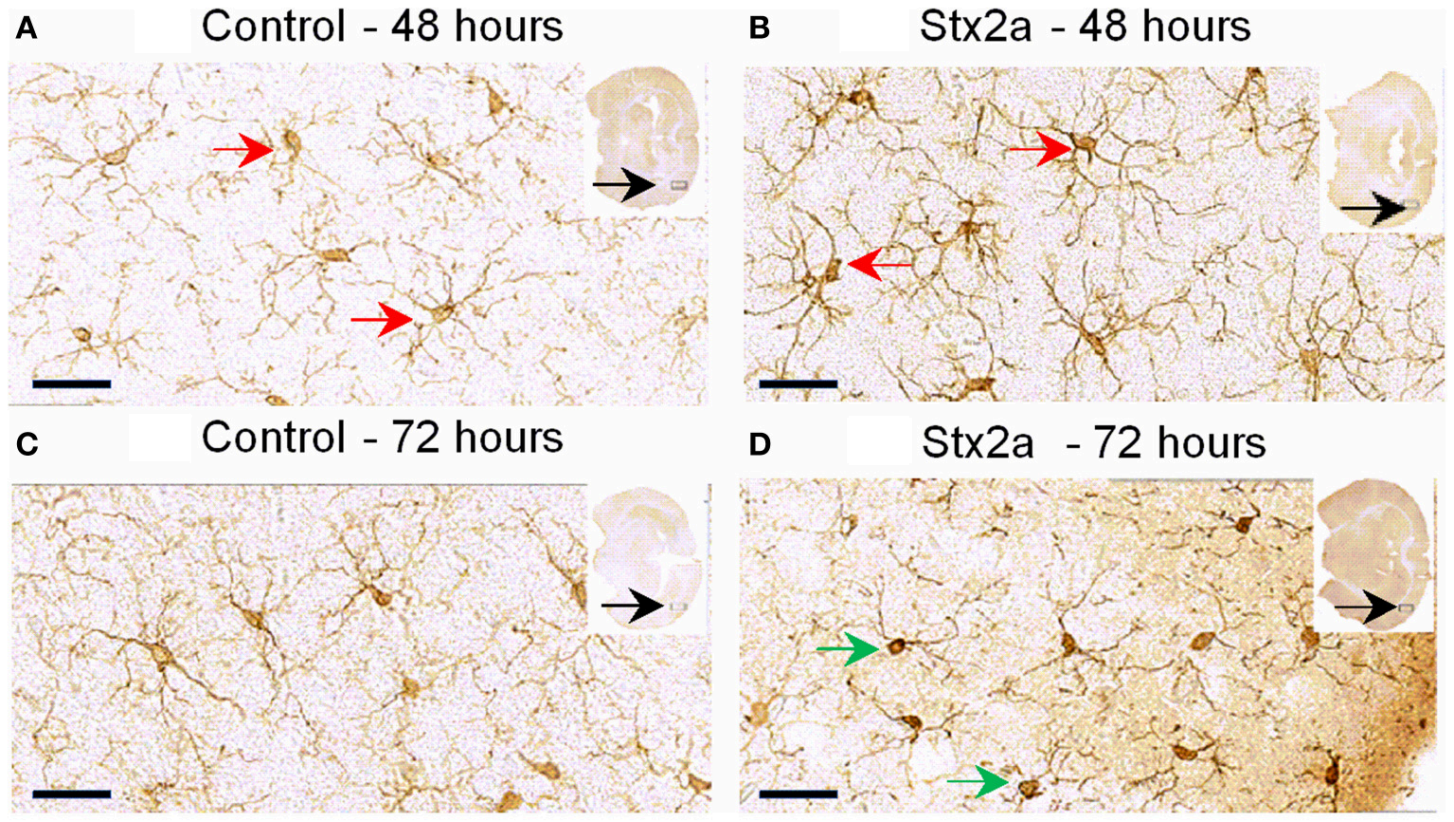
**Mouse brain temporal lobe of the cortex region stained for the microglial marker, Iba1: Cross sections of brain tissue injected with PBS control (A,C), and Stx2a (7 ng) B,D; (A,B), 48 h, (C,D) 72 h post-injection**. Compared to controls at 48 h, brains of mice injected with Stx2a show an increase in the intensity of Iba1 staining of microglial cell bodies and processes (red arrows). At 72 h post-injection with Stx2a, morphologic change from the ramified (resting stage) characterized by long branching processes, to the activated macrophage-like globular structure displaying few processes and a more intensely stained cell body (**D**, green arrows) is observed. Scale bar represent 50 μm. Insert, entire brain scan where orange arrow indicates approximate position of the magnified image (*n* = 4).

Microglial cell responses are early indicators of neural injury. These responses include up-regulation of surface-marker expression and stereotypical morphological changes, which include conversion from the resting, ramified form (characterized by numerous, long branching processes and a small cellular body), to the macrophage phagocytic form (characterized by a large, ameboid shape, with few processes). We used Iba1, the most commonly used marker to stain for microglial activation (Hoogland et al., 2015). Compared to PBS controls (Figure 4), mice injected with Stx2a showed increased Iba1 staining, and mild morphological changes associated with microglial activation (thickening of processes), which was more pronounced at 72 h compared to 48 h.

**Figure 5:**
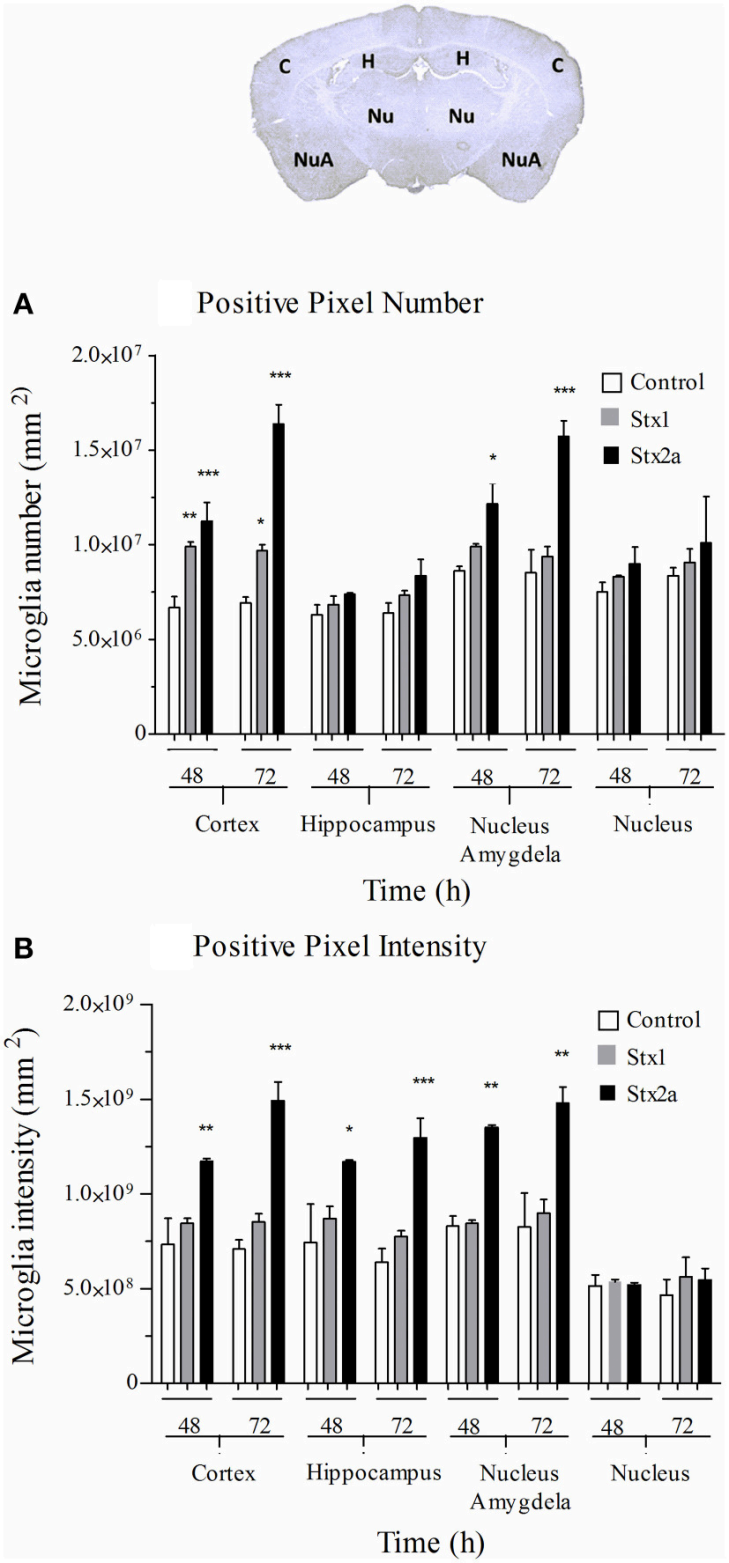
**Quantification of microglial Iba1 staining**. Pixel count in the indicated regions of the brain (top) of mice in Figure 4: injected with PBS (open bars); 1500 ng Stx1 (gray bars); or 7 ng Stx2a (black bars) at 48 and 72 h post-injection. **(A)** total positive pixel count (corresponding to microglial cell numbers). Compared to controls, for Stx2a treated mice statistically significant increases in the total pixel numbers were seen in the cortex and nucleus amygdela at 48 and 72 h. **(B)** total pixel intensity (indicative of microglial activation). Statistically significant increases in pixel intensity were seen for Stx2a at both 48 and 72 h throughout the brain with exception to the nucleus. Aperio Imagescope v12 software was used to annotate areas of interest and perform image analysis. (*n* = 4). *P*-values were ^*^P < 0.05; ^**^P < 0.04; and ^***^P < 0.02.

Digital imaging was used to quantify changes over larger and more diverse areas of the brain. The data was examined in two different ways. Positive pixel count assesses signal above background, corresponding to the area occupied by microglial cells without regard to intensity. Total pixel intensity indicates the amount of IbaI expression, and corresponds to the level of microglial activation. Stx2a-treatment resulted in statistically significant increased positive pixel numbers in the cortex and nucleus amygdala at both 48 and 72-h post-injection (Figure 5A). Significantly increased intensities were seen in all areas of the brain, except the nucleus at 72-h post-injection (Figure 5B). Stx1 only caused statistically significant increases in positive pixel numbers for the cortex (Figure 5A).

### Magnetic resonance imaging and spectroscopy

The brains of control mice and mice given a sublethal dose of Stx2a (3 ng) were examined by MRI. Brain chemistry changes upon death. Mice treated with Stx2a can deteriorate rapidly, and a sublethal dose of toxin was used to ensure viability of the animals; however all of the mice were symptomatic at the time of imaging, as evidenced by weight loss and/or lethargy. Brain scans from both the control mice and mice treated with Stx2a were unremarkable and the Stx2a treated animals did not exhibit hyperintensities in the thalamus or any other regions of the brain (data not shown), consistent with the lack of obvious pathologic changes in the H&E stained sections.

**Figure 6:**
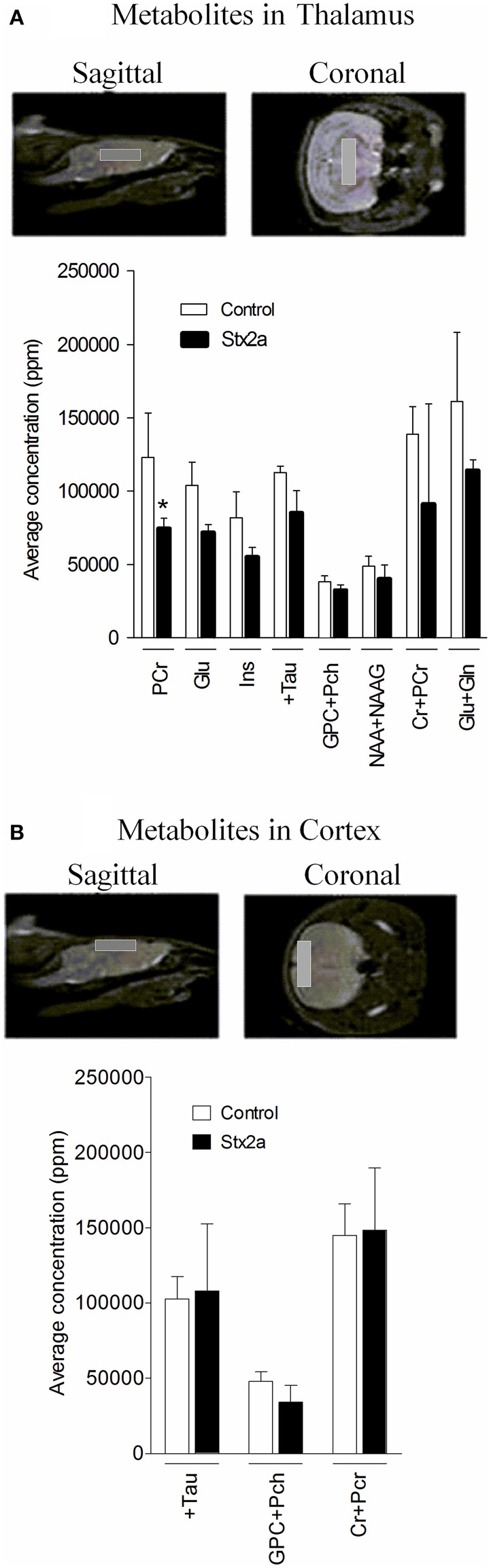
**Concentrations of brain metabolites**. MRI is shown indicating approximate placement of the sagittal and coronal voxel for thalamus **(A)** and cortex **(B)**. Average metabolite concentrations plotted as mean ± *SD* for control, PBS injected (*n* = 3 mice) and 3 ng Stx2a-injected (*n* = 4 mice). Metabolites detected include: PCr, phosphocreatine; Glu, glutamic acid; Ins, myo-inositol; + Tau, taurine; GPC + Pch, choline-containing compounds; NAA + NAAG; N-acetylaspartylglutamate + N-acetylaspartate; Cr + PCr, creatine + phosphocreatine; and Glu + Gln, glutamic acid + glutamine. Two tailed Student's *t*-test was used to assess statistical significance (^*^P < 0.03).

Brain metabolite concentrations in the thalamus and cortex regions were determined by MRS, and values within the limit of detection and with an acceptable range of standard deviation are plotted (Figure 6). In the thalamus, the Stx2a-treated mice had lower concentrations of all of the metabolites, but only phosphocreatine reached statistical significance (Figure 6A, PCr). No significant differences were seen in the metabolites of the cortex (Figure 6B).

### Brain endothelial cells are not susceptible to Stx

**Figure 7:**
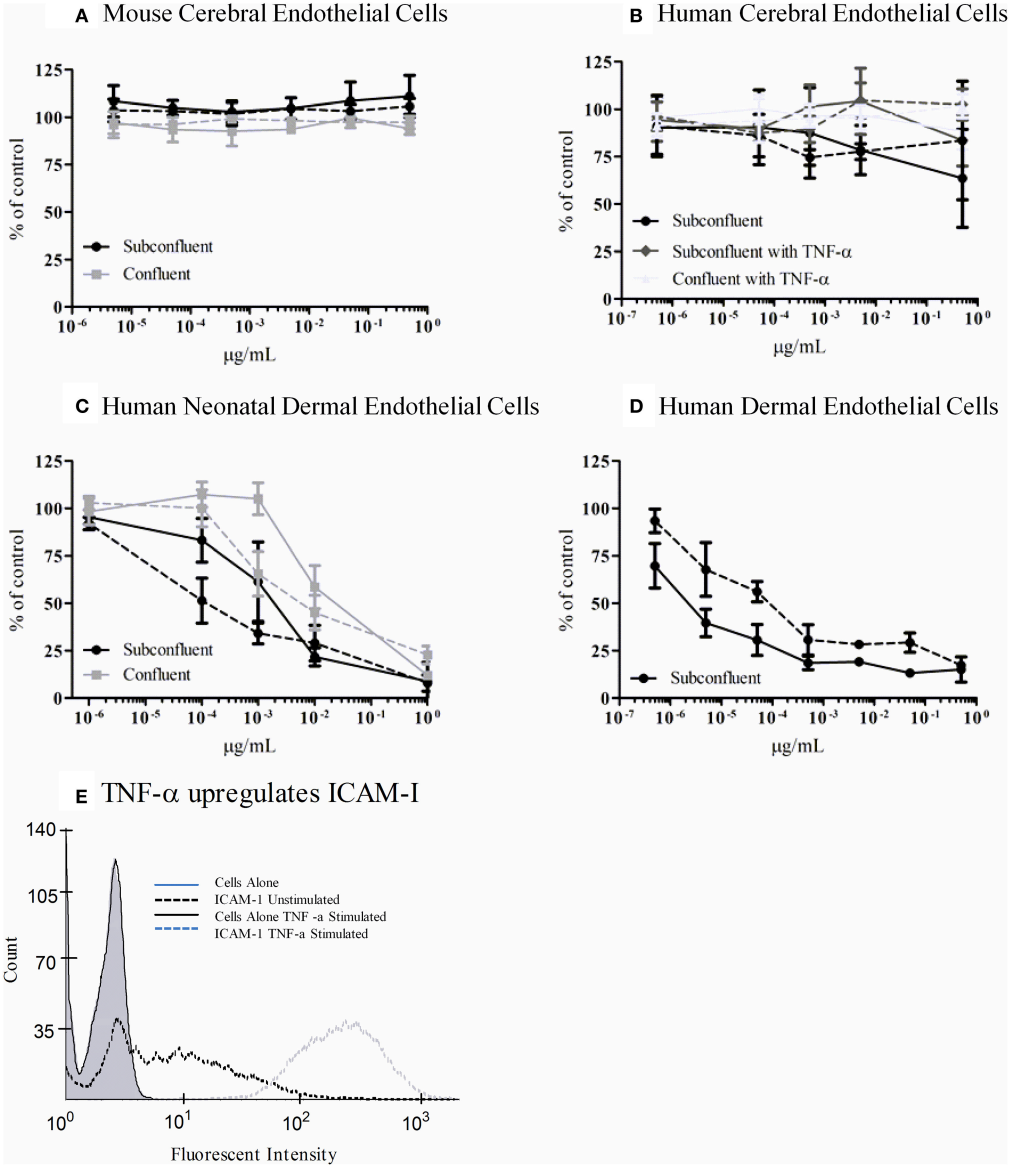
**Metabolic activity of Stx-treated microvascular endothelial cells**. bEnd.3 immortalized mouse cerebral cortex microvascular endothelial cells **(A)**, primary human cerebral cortex microvascular endothelial cells **(B)**, primary human neonatal dermal microvascular endothelial cells **(C)**, and CDC.HMEC-1 immortalized human dermal microvascular endothelial cells **(D)** were incubated with Stx1 (solid lines) or Stx2a (dashed lines) for 42 h. The toxin containing media was removed and fresh media containing 10% alamarBlue was added. Cells were incubated for an additional 3 h and the fluorescent reduction of alamarBlue was measured every 30 min. The 1 h time point is shown except for subconfluent BMECs which depicts the 3 h time point. Graphs depict toxin-treated cells as a percent of untreated control cells. Results are the average of three individual experiments and error bars correspond to standard deviation of the mean. TNF-α upregulates surface ICAM-1 **(E)**. Human brain endothelial cells were incubated with 10 ng/ml TNF-α for 24 h, stained for surface expression of ICAM-1 (CD54) and analyzed by FACS.

We tested an immortalized line, bEnd.3, from BALB/c mice and primary human microvascular endothelial cells (HBMECs) isolated from the cerebral cortex for sensitivity to Stx1 and Stx2a. Cells were incubated with 10-fold dilutions of purified Stx1 or Stx2a, ranging in concentration from 0.5 to 5 × 10^−6^ μg/ml for bEnd.3 cells or 0.5 to 5 × 10^−7^ μg/ml for HBMECs. After 42 h, mitochondrial metabolic activity was assessed by the reduction of resazurin to the fluorescent resorufin. No loss of metabolic activity was seen in the toxin-treated cells for either the mouse bEnd.3 immortalized line (Figure 7A) or the primary human brain endothelial cells (Figure 7B). Previous reports suggested that sub-confluent cells are more sensitive to Stx than confluent cells (Obrig et al., 1988), however in our studies the level of cellular density did not alter susceptibility (Figures 7A,B). In addition, previous studies reported pre-treatment with TNF-α can sensitize cells to Stx2a (Ramegowda et al., 1999; Eisenhauer et al., 2001). Pre-incubation of human brain endothelial cells with TNF-α (10 ng/ml) did not sensitize the cells to the effects of Stx (Figure 7B). However, the endothelial cells were responding to TNF-α, as evidenced by up-regulation of cell-surface expression of ICAM-1, following treatment with TNF-α (Figure 7E). Both primary neonatal dermal microvascular endothelial cells (dHMEC; Figure 7C) and immortalized human dermal microvascular endothelial cells (CDC.HMEC-1; Figure 7D) showed a dose-dependent reduction in metabolic activity following treatment with Stx1 or Stx2a, confirming the activity of both toxins.

### Stx resistance correlates with lack of Gb3 expression

To determine the molecular basis for the lack of response to Stx, flow cytometry was used to determine if both the mouse and human cells were resistant to the effects of Stx due to a lack of Gb3 receptor expression. For the bEnd.3 cells, equivalent staining was seen in the presence or absence of the primary antibody to Gb3, suggesting the mouse cells lacked expression of Gb3 (Table 1). Slightly increased values of the geometric mean fluorescence were observed for the human BMECs both without and with TNF-α stimulation. Mean fluorescence for bEnd.3 cells or HBMECs stained for Gb3 was not significantly greater than unstained control cells (*P* > 0.05 paired *t*-test). CDC.HMEC-1 cells expressed Gb3 in agreement with the susceptibility of these cells to toxin (Table 1).

**Table 1:** **Cell-surface Gb3 on cerebral cortex microvascular endothelial cells**.

	**Geometric mean fluorescent intensity**	
**Cell type**	**Secondary antibody alone**	**Gb3 and secondary antibody**
bEnd.3	12.1 ± 0.4	12.4 ± 0.1
Human brain endothelial cells	16.4 ± 1.4	18.7 ± 0.5
Human brain endothelial cells with TNF-α	16.4 ± 2.1	20.7 ± 1.7
CDC.HMEC-1	7.1 ± 1.5	29.8 ± 1.9

*Results are reported as average and standard deviation for three experiments, except CDC.HMEC-1 and TNF-α treated (n = 2)*.

## Discussion

Injection of purified Stx is sufficient to induce neurologic symptoms in experimental animals, suggesting bacterial infection is not needed. Neurologic symptoms in human disease can include lethargy, irritability, cortical blindness, and seizures (Sheth et al., 1986; Hahn et al., 1989; Nathanson et al., 2010). Mice injected with purified Stx may display hind limb paralysis, lethargy, shivering, abnormal gait and spasm-like seizures (Obata et al., 2008). Baboons exhibit seizure episodes which progressed to coma and death (Siegler et al., 2001, 2003). While the neurologic responses to Stx can be dramatic, the overall changes in the brain are mild. The goal of this study was to develop a framework to understand how mild alterations in the brain can lead to potentially life-threatening neurologic responses.

Neurologic symptoms are most commonly observed following infection with strains that produce Stx2a, not Stx1. Seven ng of Stx2a resulted in highly reproducible weight loss and severe kidney histology (Figure 2), while 1500 ng of Stx1 was much less toxic. Increased expression of the microglial activation marker Iba1 was observed for both toxins, but was more pronounced in mice given the much smaller dose of Stx2a (Figure 5A). It is likely that neurologic damage is infrequently observed following infection with *E. coli* strains that only produce Stx1 because sufficiently high doses of Stx1 are not easily achieved *in vivo*.

Stx can promote disease by different mechanisms. Internalized Stx cleaves the ribosomal RNA, causing protein synthesis inhibition. In some cells, protein synthesis inhibition activates apoptotic death pathways, resulting in death of the Stx-treated cells (Jones et al., 2000; Ching et al., 2002; Fujii et al., 2003). Several cell types in the kidney are known to be killed by Stx (van Setten et al., 1997; Hughes et al., 1998; Fuller et al., 2011; Dettmar et al., 2014), and we observed extensive cellular necrosis and damage to the kidneys of the Stx2a-treated mice. No hyperintensities were observed by MRI in the brains of living mice, indicating that unlike the kidney, massive cellular death is not the mechanism by which Stx-induces brain damage.

In other cells, Stx-mediated inhibition of protein synthesis can activate cellular stress responses without causing death (Iordanov et al., 1997; Foster and Tesh, 2002; Smith et al., 2003). Activation of stress responses can alter the behavior of the toxin-susceptible cell, as well as cells that are resistant to the action of Stx. For example, macrophages respond to Stx by producing pro-inflammatory cytokines (Tesh et al., 1994; Harrison et al., 2005), which can induce global systemic responses. Neurologic damage could result from activation of cellular stress responses, either directly in the cells of the brain or in response to mediators produced elsewhere in the body, or both. While some studies have reported upregulation of stress responses by endothelial cells treated with Stx (Matussek et al., 2003; Petruzziello-Pellegrini et al., 2012), in our studies, both a mouse brain endothelial cell line and primary human brain endothelial cells were resistant to Stx1 and Stx2a under all conditions tested. It has long been known that primary endothelial cells vary in their sensitivity to Stx and TNF-α (Louise and Obrig, 1991). The published literature differs on the inherent sensitivity of human BMECs. Studies using commercially available cells have reported HBMECs to be fairly resistant to Stx (Hughes et al., 2002; Stricklett et al., 2002), as we report using cells obtained from Cell Systems Corporation. However, two studies reported HBMECs to be highly sensitive to Stx using cells they isolated (Fujii et al., 2008; Bauwens et al., 2010). It is difficult to determine why inconsistent results are seen.

One approach to identify cells that are altered, but not killed by Stx, has been to focus on the cells that express the Stx receptor, Gb3. While not all cells that express Gb3 are susceptible to Stx (Storck et al., 2012), cells that lack Gb3 expression are resistant, and mice lacking Gb3 expression are completely protected from Stx-mediated death (Okuda et al., 2006). Porubsky et al. (2014) found that when Gb3 expression was selectively deleted only in renal tubule cells, about 65% of mice still succumbed to neurological complications. Furthermore, when Gb3 expression was deleted from both renal tubule cells and endothelial cells, survival only increased by 25%. These results suggest that neurologic damage is not just a secondary response to kidney damage, and Gb3 expressing cells which do not reside in the kidney or endothelium play a significant role in Stx mediated complications and death.

Neurons within the mouse olfactory bulbs, cerebral cortex, hippocampus, striatum, amygdale, cerebellum, hypothalamus, thalamus and medulla oblongata stain positive for Gb3 (Obata and Obrig, 2010). Obata et al. (2008) observed that Gb3 positive motoneurons could also be labeled with Stx2a, and ultrastructural studies of the brains of Stx-treated mice and rats exhibited neuronal damage, including demyelinated axons, cytoplasmic edema and degenerative phenotypes (Goldstein et al., 2007; Obata et al., 2008; Tironi-Farinati et al., 2013). However, mouse astrocytes, which are reported to lack Gb3 expression, exhibit severe swelling and cellular breakdown following Stx injection (Obata et al., 2008; Tironi-Farinati et al., 2013), suggesting these cells are indirectly damaged following Stx challenge. In electron microscopy, lamellipodia-like projections from either reactive astrocytes or glial cells which lack Gb3 expression were observed interrupting the pre- and post-neuronal synapses in the brains of Stx-treated mice (Obata et al., 2008; Tironi-Farinati et al., 2013). Obata et al. (2008) put forward a model where Stx stimulates excessive neurotransmitter release. Since this is toxic to neurons, astrocytes attempt to limit damage by occluding the synapse with the inserted lamellipodium and by taking up excess neurotransmitter. This model explains how damage to one cell (the neuron) can cause responses in other cells, how loss of motor control could occur in the absence of gross pathology, and why the neurologic damage appears to be reversible in humans.

While neurons can be a target for Stx, and Stx2 has been immunolocalized in the brain parenchyma of mice (Armstrong et al., 2006; Obata et al., 2008), Stx must bypass the blood brain barrier to gain access to these cells. In studies tracking the distribution of radio-labeled Stx in mice following intravenous injection, at early time points neither Stx1 nor Stx2a were detected in the central nervous system or brain stem of the animals (Rutjes et al., 2002). However, at 48 h Stx2a, but not Stx1, was detected in the brain (Armstrong et al., 2006). These results suggest that there is a lack of immediate binding of Stx2 to endothelial cells *in vivo*, consistent with our studies that do not support a role for Stx acting directly on brain endothelial cells. However, over time Stx2 can access the brain tissues, presumably requiring disruption of the blood brain barrier. This could result as a secondary response following damage to other organ systems. For example, pro-inflammatory responses can alter the blood brain barrier. Alternatively, energy depletion by lack of glucose and oxygen intake or electrolyte disorders could damage the brain. The reduced levels of phosphocreatine, a high-energy phosphate donor, seen by MRS in this study indicate increased metabolic activity in the Stx2a-treated mice.

Overall, our studies are consistent with the hypothesis that systemic responses to Stx alter the blood brain barrier, allowing access to the neurons. Our observation of red blood cell congestion in both the kidney and brain is consistent with vascular involvement, even though the brain endothelial cells may not be a direct target for Stx early in disease. Furthermore, symmetrical microglial activation occurring in many parts of the brain suggests that local ischemic or hemorrhagic events are not responsible for the neurologic damage, and a more global process is operating. A more detailed understanding of the systemic alterations induced by Stx could allow us to develop therapeutic approaches to treat this currently untreatable disease.

## Author contributions

Conceived and designed the experiments: AW. Performed the experiments: SP, CP, KM. Analyzed the data: AW, SP, CP, KM. Contributed reagents/materials/analysis tools: AW, DC. Contributed to the writing of the manuscript: AW, SP, CP, KM.

### Conflict of interest statement

The authors declare that the research was conducted in the absence of any commercial or financial relationships that could be construed as a potential conflict of interest.
